# Supplementary material for: Cross-kingdom signalling regulates spore germination in the moss Physcomitrella patens
Source: Sci Rep. 2020 Feb 13;10:2614. doi: 10.1038/s41598-020-59467-5 (PMC7018845; doi:10.1038/s41598-020-59467-5)
Supplement: Supplementary file 1 — Supplementary Information. [file 41598_2020_59467_MOESM1_ESM.pdf]

Supplemental material for:

Cross-kingdom signalling regulates spore germination in the moss *Physcomitrella patens*

Eleanor F. Vesty[1,2], Amy L. Whitbread[1,3+], Sarah Needs[1,4+], Wesal Tanko[1+], Kirsty Jones[1+], Nigel Halliday[5], Fatemeh Ghaderiardakani[1], Xiaoguang Liu[5,6], Miguel Cámara[5*], Juliet C. Coates[1*].

[1] School of Biosciences, University of Birmingham, Edgbaston, Birmingham, UK.

[2] University Centre Shrewsbury, Guildhall, Frankwell Quay, Shrewsbury, Shropshire, UK

[3] Karlsruhe Institute of Technology, Karlsruhe, Baden-Württemberg, Germany

[4] School of Life, Health and Chemical Sciences, Open University, Walton Hall, Kents Hill, Milton Keynes, UK

[5] National Biofilm Innovations Centre, University of Nottingham Biodiscovery Institute, School of Life Sciences, University of Nottingham, University Park, Nottingham, UK.

[6] Institute of Life Sciences, Jiangsu University, Zhenjiang, China

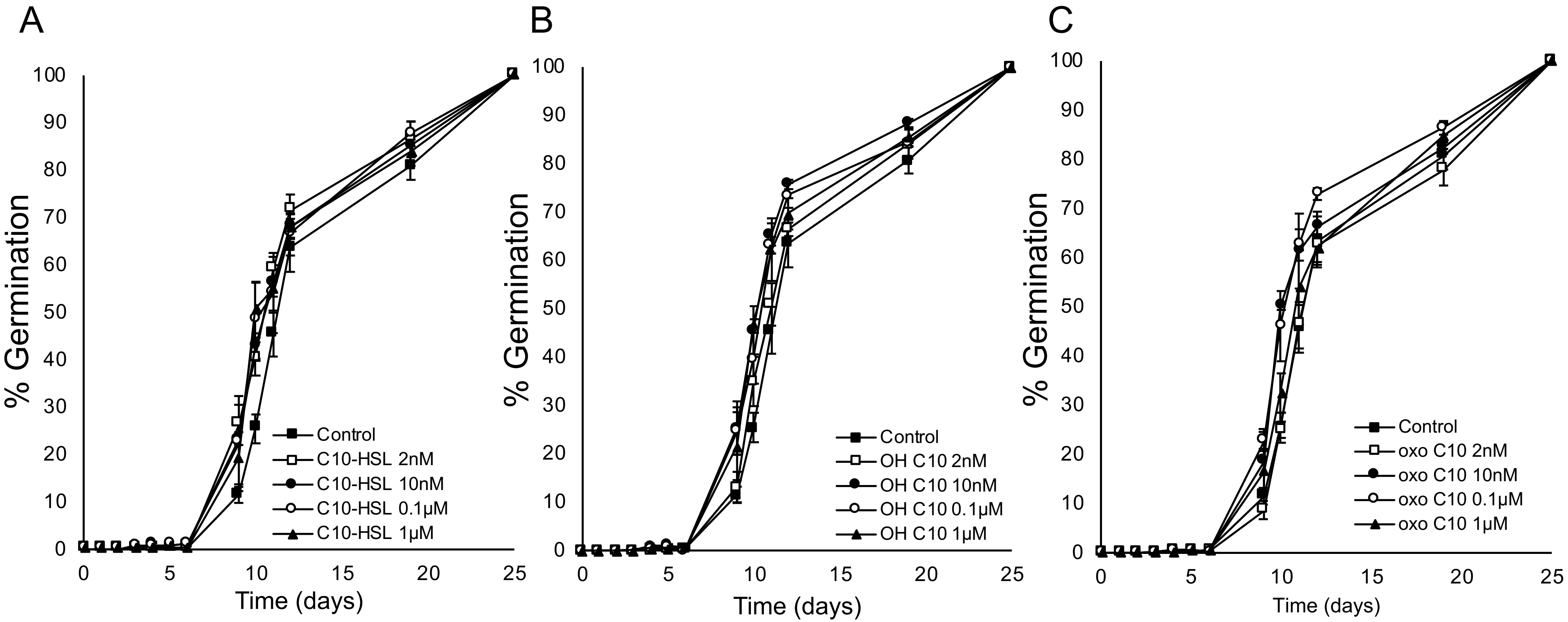

Supplemental Figure 1. Effect of side group substitution on AHL activity (C10 chain length) towards *Physcomitrella* spore germination.

A) Unsubstituted C10-HSL, where lower concentrations (2-10nM) promote germination more potently, B) 3-OH-C10-HSL (more potent promotion of germination at 10nM, C) 3-O-C10-HSL (more potent germination promotion at 0.1μM).

These data are summarized in Figure 3B.

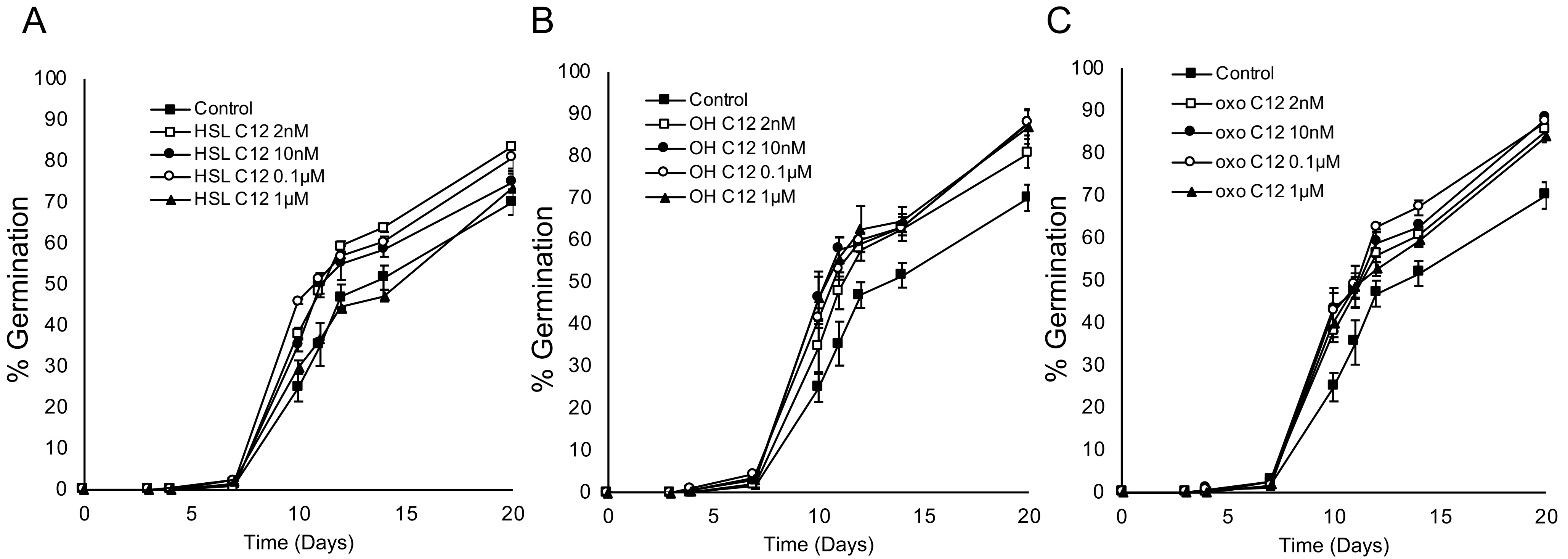

Supplemental Figure 2. Effect of side group substitution on AHL activity (C12 chain length) towards *Physcomitrella* spore germination.
A) Unsubstituted C12-HSL (most potent promotion of spore germination at 2nM), B) 3-OH-C12-HSL (similar promotion of spore germination over the whole range of concentrations), C) 3-O-C12-HSL (more potent promotion of spore germination at 1μM). These data are summarized in Figure 3B.

AHL NAME	STRUCTURE
C4-HSL	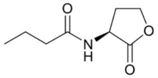
C6-HSL	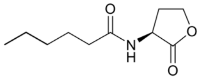
C8-HSL	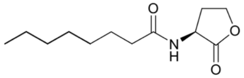
C10-HSL	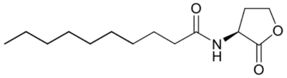
C12-HSL	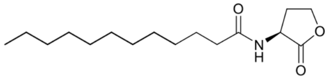
3-O-C4-HSL	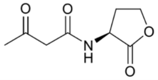
3-O-C6-HSL	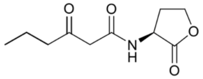
3-O-C8-HSL	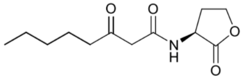
3-O-C10-HSL	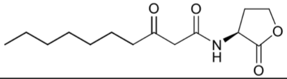
3-O-C12-HSL	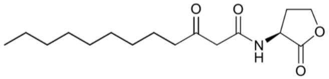
3-OH-C4-HSL	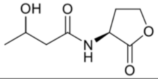
3-OH-C6-HSL	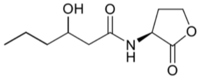
3-OH-C8-HSL	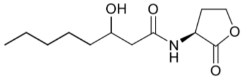
3-OH-C10-HSL	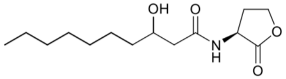
3-OH-C12-HSL	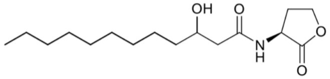

Supplemental Table 1. AHLs used in this study.
